# Extra-Esophageal Pepsin from Stomach Refluxate Promoted Tonsil Hypertrophy

**DOI:** 10.1371/journal.pone.0152336

**Published:** 2016-04-08

**Authors:** Jin Hyun Kim, Han-Sin Jeong, Kyung Mi Kim, Ye Jin Lee, Myeong Hee Jung, Jung Je Park, Jin Pyeong Kim, Seung Hoon Woo

**Affiliations:** 1 Biomedical Research Institute, Gyeongsang National University Hospital, Jinju, Gyeongsangnamdo, Korea; 2 Department of Otorhinolaryngology-Head and Neck Surgery, Sungkyunkwan University School of Medicine, Samsung Medical Center, Seoul, Korea; 3 Department of Otolaryngology, Gyeongsang National University Hospital, Jinju, Gyeongsangnamdo, Korea; 4 Institute of Health Sciences, Gyeongsang National University Hospital, Jinju, Gyeongsangnamdo, Korea; Universitatsklinikum Freiburg, GERMANY

## Abstract

**Background:**

Gastroesophageal reflux is associated with numerous pathologic conditions of the upper aerodigestive tract. Gastric pepsin within reflux contributes to immunologic reactions in the tonsil. In this study, we aimed to find the relationships between pepsin and tonsillar hypertrophy.

**Methods and finding:**

We explored the notion whether tonsillar hypertrophy was due to pepsin-mediated gastric reflux in tonsil hypertrophy. Fifty-four children with tonsil hypertrophy and 30 adults with tonsillitis were recruited before surgical treatment. Blood and tonsil tissues from each patient were harvested for analysis of changes in lymphocyte and macrophage numbers coupled with histological and biochemical analysis. Pepsin was expressed at different levels in tonsil tissues from each tonsillar hypertrophy. Pepsin-positive cells were found in the crypt epithelium, surrounding the lymphoid follicle with developing fibrosis, and also surrounding the lymphoid follicle that faced the crypt. And also, pepsin staining was well correlated with damaged tonsillar squamous epithelium and TGF-β1 and iNOS expression in the tonsil section. In addition, pepsin and TGF-β1-positive cells were co-localized with CD68-positive cells in the crypt and surrounding germinal centers. In comparison of macrophage responsiveness to pepsin, peripheral blood mononuclear cells (PBMNCs) were noticeably larger in the presence of activated pepsin in the child group. Furthermore, CD11c and CD163-positive cells were significantly increased by activated pepsin. However, this was not seen for the culture of PBMNCs from the adult group.

**Conclusions:**

The lymphocytes and monocytes are in a highly proliferative state in the tonsillar hypertrophy and associated with increased expression of pro-inflammatory factors as a result of exposure to stomach reflux pepsin.

## Introduction

Tonsil hypertrophy is currently the most common reason for tonsillectomy. Enlargement of the tonsils occurs due to an absolute increase in the total number of lymphocytes in the tissue and resulting in an increase in tissue volume.[1, 2] The precise mechanism by which lymphocyte stimulation and proliferation occurs has yet to be determined. It has been inferred that antigenic stimulation of the tissue lymphocytes leads to an increase in lymphocytic number and activity. Previous attempts at identifying the pathophysiologic mechanisms have focused on microbiological and immunologic changes in enlarged tonsils. Many studies have reported a possible role of bacterial organisms in the pathogenesis of tonsil hypertrophy.[3–5] However an increase in the absolute number of lymphocytes within the tonsils without a clinical infection was previously shown[2], and the specific antigens responsible for these changes have not been identified.

Recent research has looked at the relationship between extraesophageal reflux disease and upper airway disorders. Chronic sinusitis[6], otitis media with effusion[7–9] and laryngeal disorders have all been studied with possible etiologic links to extraesophageal reflux.[10, 11] Refluxate contains gastric enzymes (pepsin and HCl) as well as duodeno-pancreatic enzymes (bile acids and trypsin). The role of pepsin in gastric juice as part of the refluxate and interaction with otolaryngology systems and in ENT organs (ear, nose, and throat) has been extensively studied.[1, 10, 11]

Pepsin is an enzyme converted from pepsinogen, which is produced by the chief cell of the stomach, and plays an important role in digestion. Normally, pepsin is only found in the stomach contents. However, if extraesophageal reflux occurs, the stomach contents from the reflux can reach the laryngopharynx, and pepsin as part of the stomach reflux can be detected in the laryngopharyngeal areas. This is indeed the case as Johnston et al.[12] reported that pepsin was detected in upper airway mucosa and induces a proinflammatory cytokine cycle, resulting in inflammatory damage to the laryngeal mucosa. These data have suggested a role for refluxed pepsin in the breakdown of the immune defense mechanism in the mucosal or epithelial linings and promotion of inflammatory causing agents.[12–15]

Similarly for the tonsil, we hypothesized that gastric pepsin within reflux has a key role in its immunologic reaction of tonsil and that pepsin exposure induces tonsil hypertrophy. In this study, we aimed to find the relationship between gastric pepsin and tonsillar hypertrophy.

## Materials and Methods

### Ethics statement

This study was approved by the Gyeongsang National University Hospital Institutional Review Board (# GNUHIRB-2014-02-006). Written Informed consent was obtained from the all patients (or parents) prior to their inclusion in the study.

### Study subjects

This study was performed on 84 patients with the clinical diagnosis of tonsillar hypertrophy. They visit our hospital for the removal of tonsil because they suffer from chronic inflammation at tonsil or snoring/sleep apnea due to tonsil enlargement. All patients underwent physical examination to confirm the diagnosis of tonsillar hypertrophy or chronic tonsillitis. The mean size of tonsil was grade 2.5 in tonsil hypertrophy group and grade 1.0 in chronic tonsillitis group. (Table 1). We obtained the tonsil tissues from 54 children prior to their surgical treatment (41 boys and 13 girls; age range 4–16 years, mean age 8 years) and from 30 adults (17 males and 13 females; age range 17 and over, mean age 29 years). Patients with systemic disorders and other clinical problems were not included in this study. Whole tonsillectomy was performed under general anesthesia using dissection method and inferior part of tonsil was selected for tissue sampling. None had any postoperative complication.

**Table 1:** Characteristics of patients.

	Tonsil hypertrophy group (N = 54)	Chronic tonsillitis group (N = 30)
**Age–yr**		
** Mean**	**8.33**	**29.25**
**Median**	**8.0**	**29.0**
**Gender—no. (%)**		
**Male**	**41**	**17**
**Female**	**13**	**13**
**Mean tonsil size–Grade**	**2.5**	**1.0**

*Grading Scale

A. Tonsil 0: Tonsils fit within tonsillar fossa

B. Tonsil 1+: Tonsils <25% of space between pillars

C. Tonsil 2+: Tonsils <50% of space between pillars

D. Tonsil 3+: Tonsils <75% of space between pillars

E. Tonsil 4+: Tonsils >75% of space between pillars.

### Protein preparation and immunoblot analysis

Tissue extracts from tonsils were prepared as follows: tonsils were removed and homogenized in lysis buffer made up of PBS (pH 7.4), 1% Triton X-100, 1 mM EDTA containing 10 μM leupeptin and 200 μM phenylmethylsulfonyl fluoride. The lysates were sonicated several times for 3 to 5 min each and centrifuged at 12,000 rpm for 20 min at 4°C. The supernatants were collected and the protein concentration of each lysate was determined using a bicinchoninic acid (BCA) protein assay kit (Pierce, Rockford IL, USA) according to the manufacturer’s protocol. Bovine serum albumin was used as a standard. Equal amounts of protein (50 μg) were loaded on to a 10% sodium dodecyl sulfate (SDS) polyacrylamide gel. After electrophoresis, proteins in the gel were transferred onto a nitrocellulose membrane (Schleicher & Schuell, Dassel, Germany). Membranes were blocked with 5% non-fat milk in Tris-buffered saline containing 0.1% Tween-20. Blots were probed with primary antibodies to polyclonal anti-Pepsin A (sc-99081, Santa Cruz Biotechnology CA, USA) at 4°C overnight. As a loading control, blots were re-probed with anti-β-actin antibody (Sigma, St. Louis MO, USA). The primary antibody was visualized using secondary antibodies (horseradish peroxidase-conjugated goat anti-rabbit IgG, 1:10,000; Pierce) with an ECL kit (Amersham Pharmacia Biotech, Piscataway NJ, USA).

### Immunohistochemistry

Immunostaining was performed on 5-mm thick coronal sections of paraformaldehyde-fixed and paraffin-embedded sections using the avidin-biotinylated-horseradish peroxidase-complex kits (ABC; Vector Laboratories, Burlingame CA, USA). Following deparaffinization in xylene, sections were rehydrated with ethanol. After washing in PBS, the sections were blocked with 1% normal goat serum and then treated with an anti-Pepsin A (sc-99081, Santa Cruz), iNOS, TGF-β1, and CD68 antibodies purchased from Santa Cruz Biotechnology at 4°C overnight in a humidified chamber. After washing in PBS, they were incubated for 90 min at room temperature with secondary antibody (Santa Cruz Biotechnology, biotin-conjugated anti-rabbit immunoglobulin G, 1:200). Finally, the sections were incubated with ABC for 60 min at room temperature, rinsed in PBS, and then developed with 0.027% 3,3'-diaminobenzidine tetrahydrochloride (Sigma) with 0.003% hydrogen peroxide. The sections were counterstained with hematoxylin (Sigma).

### Double immunofluorescence staining

To characterize Pepsin A-positive cells, double immunofluorescence was performed on the tonsil tissues. Deparaffinization and antigen retrieval were performed. Non-specific antibody binding was blocked in PBS with 0.1% normal donkey serum (Vector Laboratories) and 0.3% Triton X-100 (Sigma) for 45 min. Sections were then incubated with anti-Pepsin A antibody (1:100; sc-99081, Santa Cruz) diluted in PBS containing 0.1% bovine serum albumin (Sigma) at 4°C overnight. After rinsing, donkey Cy3-conjugated anti-rabbit IgG secondary antibody (1:100; EMD Millipore, Billerica MA, USA) was applied for 1 hour at room temperature. For double labeling, after blocking in PBS containing 10% normal goat serum and 0.3% Triton X-100, sections were incubated with anti-CD68 (1:100; Santa Cruz) at 4°C overnight. Alexa488-conjugated anti-mouse IgG secondary antibody (1:100; Invitrogen, Carlsbad CA, USA) was then applied for 1 hour at room temperature. Sections were mounted with anti-fading solution containing 4',6-diamidino-2-phenylindole (DAPI) (Vector Laboratories), and observed under a fluorescence microscope (Carl Zeiss Microscopy GmbH, Jena, Germany). To characterize CD68-positive cells, double immunofluorescence was performed, as described above. For double labeling, anti-TGF-β1 (1:100; Santa Cruz) and anti-iNOS (1:100; Santa Cruz) were applied and then donkey Cy3-conjugated anti-rabbit IgG secondary antibody (1:100; EMD Millipore) on CD68-stained sections.

### Reverse transcription-Polymerase chain reaction (RT-PCR)

Total RNA was extracted from tonsil tissues using the TRIzol method according to the protocol recommended by the manufacturer (GIBCO, Grand Island, NY). Equal amounts (5 ug) of DNA-free total RNA from each sample were converted to cDNA using 200 U of SuperScript II RT (GIBCO, Grand Island, NY) in a 20 μl reaction volume. Reverse transcription was performed at 22°C for 10 min, at 42°C for 45 min, and at 95°C for 5 min. The reaction products (2.0 μl) were subjected to PCR amplification (Promega, Madison, WI, USA) in a 50 μl reaction volume. Each primer sequences were as follows: IL-1β (189 bp), 5′-TCATTGCTCAAGTGTCTGAAGC-3’ (sense) and 5′-TGGTCGGAGATTCGTAGC-3’ (antisense); IL-6 (628 bp), 5′-ATGAACTCCTTCTCCACAAGCGC-3′ (sense) and 5′-GAAGAGCCCTCAGGCTGGACTG-3′ (antisense); TNF-β (443 bp), 5′-AGTGACAAGCCTGTAGCCC-3′ (sense) and 5′-GCAATGATCCCAAAGTAGACC-3′ (antisense). PCR was performed using the BioRad thermal cycler according to the instructions provided by the manufacturer. Equal volumes of the amplification products were analyzed by 1.5% of agarose gel electrophoresis with 0.5 mg/ml of ethidium bromide staining.

### Flow cytometric analysis and *in vitro* cultivation

All blood samples were processed within 2 hours after taking blood. Peripheral blood mononuclear cells (PBMNCs) were isolated by density gradient centrifugation over a Ficoll gradient (Sigma, St. Louis MO, USA) for 25 min at 2,300 rpm and were washed three times in PBS. PBMNCs at 1×10^5^ cells were then analyzed by flow cytometry. Whether pepsin is involved in monocyte to macrophage differentiation, the remaining cells were plated on culture dishes in presence/or absence of activated pepsin (Thermo scientific, Rockford IL, USA) containing monocyte culture conditions. To identify the level of macrophage population, after 8 and 15 days, cells were harvested and assayed by flow cytometry using antibody for CD11c and CD163. All cell cultures were maintained at 37°C with 5% CO_2_ in a humidified atmosphere.

### Cell viability

RAW264.7 cells, mouse macrophage-like cell line, were cultivated to investigate the effect of pepsin on macrophage proliferation. The cells were treated in various concentration of pepsin (0.01–5 μg/ml) and cell viability was examined by CCK-8 kit (Cell Counting Kit-8, Dojindo Molecular Tech. Inc., Rockville, MD, USA). Cell viability in each concentration was represented as fold change. The fold changes are calculated as the ratio of the final value in the each presence of pepsin to the value in absence of pepsin (set as “1”). Values are represented as the mean ± SEM. *P < 0.05 vs. the corresponding absence of pepsin (0 μg/ml).

### *In vitro* migration assay

A standard monolayer scratch wound model was used to characterize the responsiveness of macrophages to pepsin. RAW264.7 cells were seeded in 6-well tissue culture plates, cultured to confluence, and the monolayers were wounded by scratching along the surface of the tissue culture plastic with a razor blade. The blade was pressed down in the middle of the dish, thus cutting the cell layer and concomitantly marking the "wound boundary" on the underlying plastic. Then the blade was gently slid unidirectionally to remove half of the confluent cell layer. The "wounded monolayer" was washed twice with phosphate-buffered saline pH 7.4 (PBS), re-fed with 1 mM mitomycin-containing serum-deprived medium, and incubated under standard culture conditions for 24 hr.

### Statistical analysis

All data are presented as mean ± S.E.M. Comparisons between groups were analyzed by two-tailed *t* test. Probability values (*P*) < 0.05 were considered significant.

## Results

### Pepsin was expressed in the tonsil

**Fig 1:**
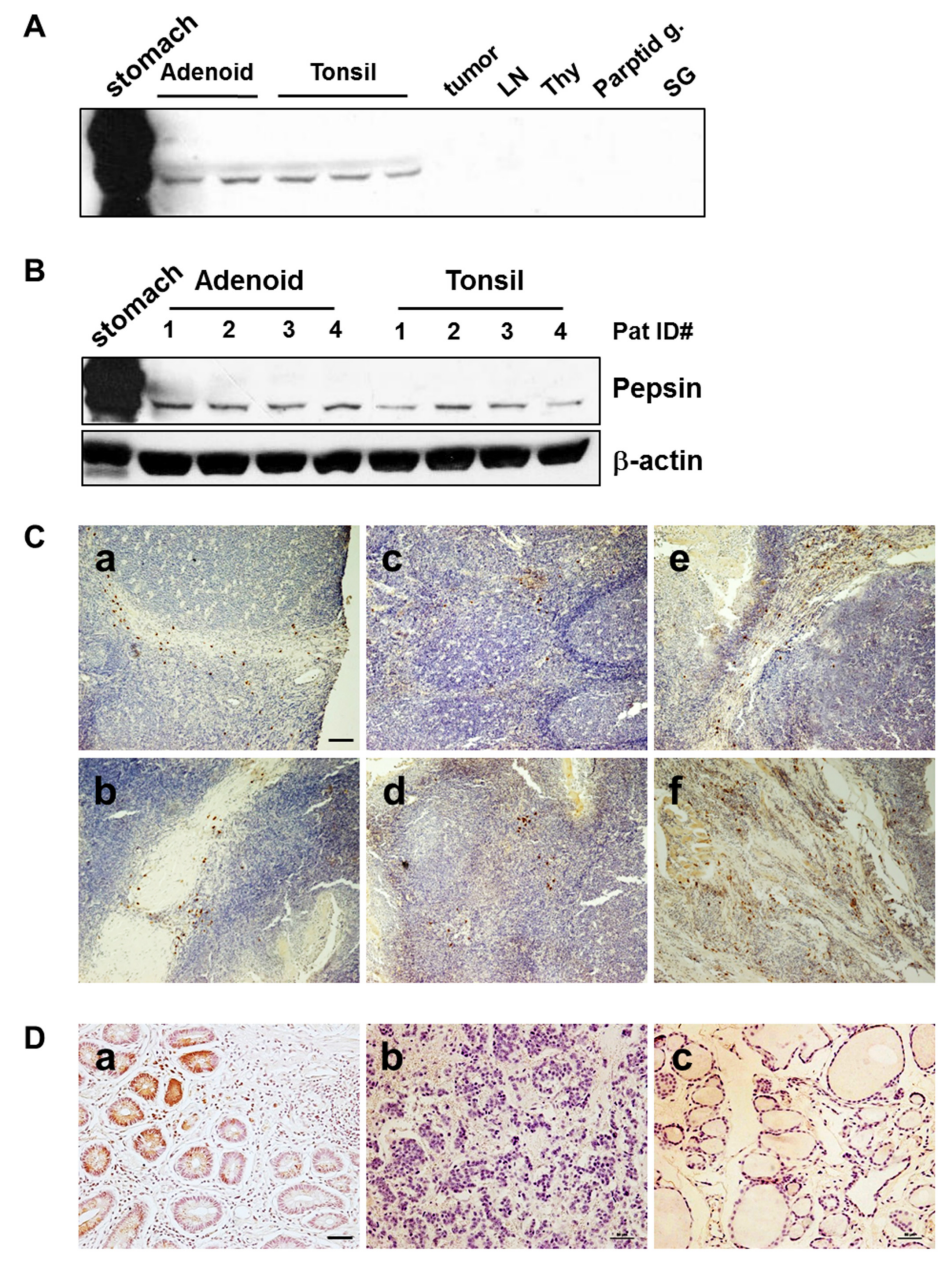
Pepsin detected in the tonsillar hypertrophy tissues. **A)** Pepsin was expressed in the tonsil tissue. Stomach tissues were used as positive control for pepsin detection. Other tissues were used as negative control. **B)** Pepsin staining levels were different from each patient. β-actin was used for housekeeping control. LN, lymph node. Thy, thyroid. Parotid g, parotid gland. SG, salivary gland. **C)** Pepsin-positive cells localized below the surface epithelium, mainly in the crypt regions (a and b), also surrounding the more negative germinal centers (c and d), and surrounding the lymphoid follicle with excessive developing fibrosis (e and f). **D)** Stomach sections showed a typical pattern of pepsin staining (a), but not in the lymph node (b) and thyroid (c).

**Fig 2:**
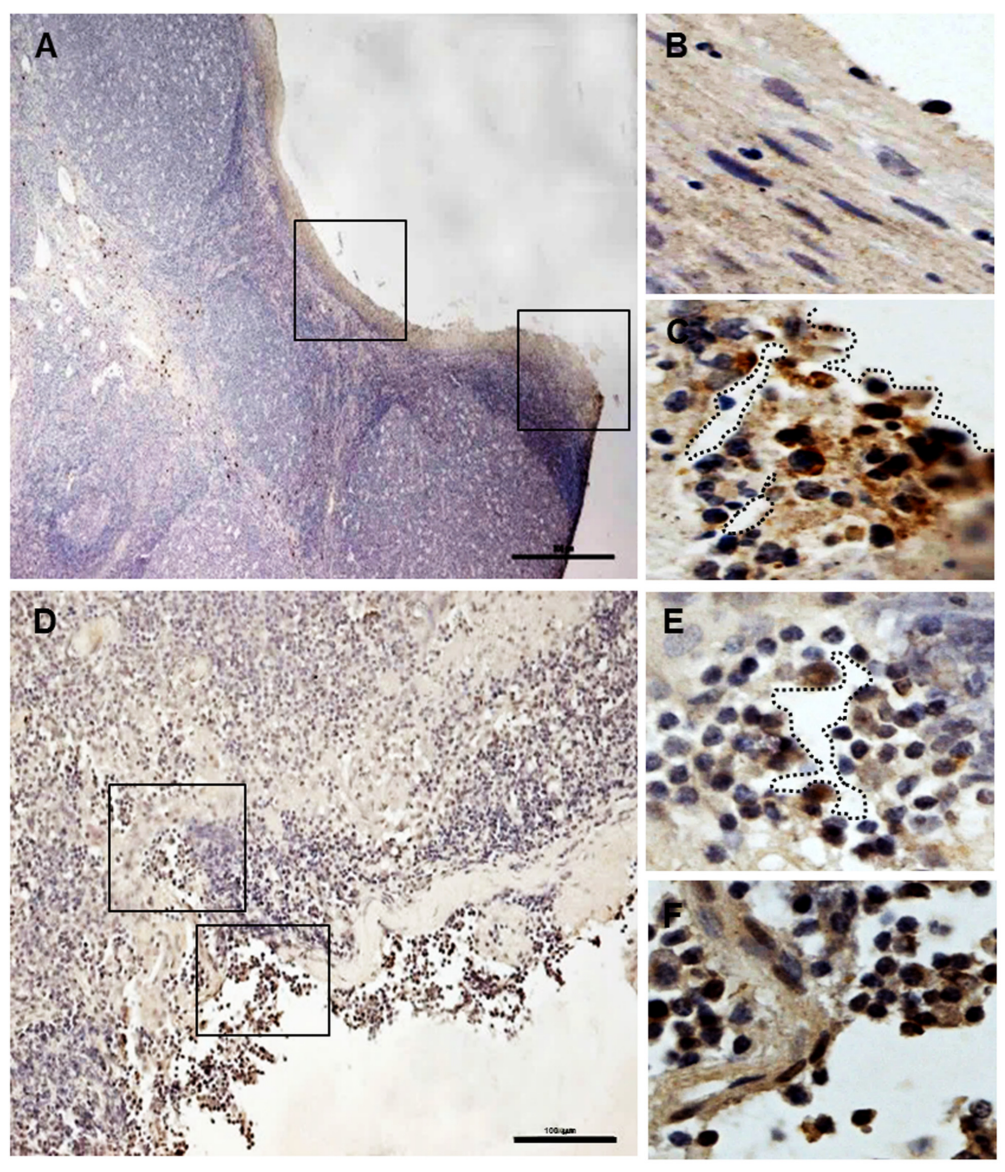
The injured tonsil site and pepsin-positive cells in the tonsil sections. No positive signals were detected in the non-injured tonsillar squamous epithelium (left insert in A and B). Pepsin-positive cells were strongly found in the damaged tonsillar squamous epithelium (right insert in A and C, D, E, and F). Dashed lines show damaged clefts and broken squamous epithelium. Inserts in A and D were magnified as “B and C” and “E and F”, respectively. Scale bar, 50 μm (A) and 100 μm (D).

Immunoblot data showed that pepsin protein was expressed as a single band from extracts of both the tonsil of patients with tonsil hypertrophy. Pepsin was highly expressed as multiple bands in the positive control of stomach tissue extracts. Virtually no pepsin staining was observed in other tissues including tumor, lymph node (LN), thyroid (Thy), parotid gland (Parotid g.), salivary gland (SG) (Fig 1A). All of tonsil tissues exhibited a positive signal for pepsin (Fig 1B). However, detection levels of pepsin in the tonsil were slightly different in each patient (Fig 1B). Immunohistochemical staining was performed to identify the pepsin-positive cells in the tonsil tissue. Pepsin-positive cells are selectively found in the below surface epithelium, mainly located in the crypt (Fig 1C-a and b), surrounding the more negative germinal centers (Fig 1C-c and d), and also surrounding the lymphoid follicle with excessive developing fibrosis (Fig 1C-e and f). Stomach sections used as a positive control showed a typical pattern of pepsin staining, predominantly in the glandular cells (Fig 2D-a), but not in the lymph node (Fig 1D-b) and thyroid (Fig 1D-c).

### The pepsin-positive cells were detected in the damaged tonsil squamous epithelium

To confirm the relation with tonsil squamous epithelium damage and reflux, we tried to find pepsin-positive cells in the injured or intact tonsillar epithelial architecture. Damaged squamous epithelium, irregular or broken, were found in the tonsil tissues with tonsillar hypertrophy (Fig 2A and 2D, below). Pepsin-positive cells were detected in the injured sites (right insert in Fig 2A, 2D, 2E and 2F) compared to normal epithelium (left insert in Fig 2A and 2B). In particular, the signals were strongly found around clefts and damaged tonsil squamous epithelium (dashed lines in Fig 2C and 2E).

### TGF-β1 and iNOS-positive cells were detected in the tonsil of patients with tonsil hypertrophy

**Fig 3:**
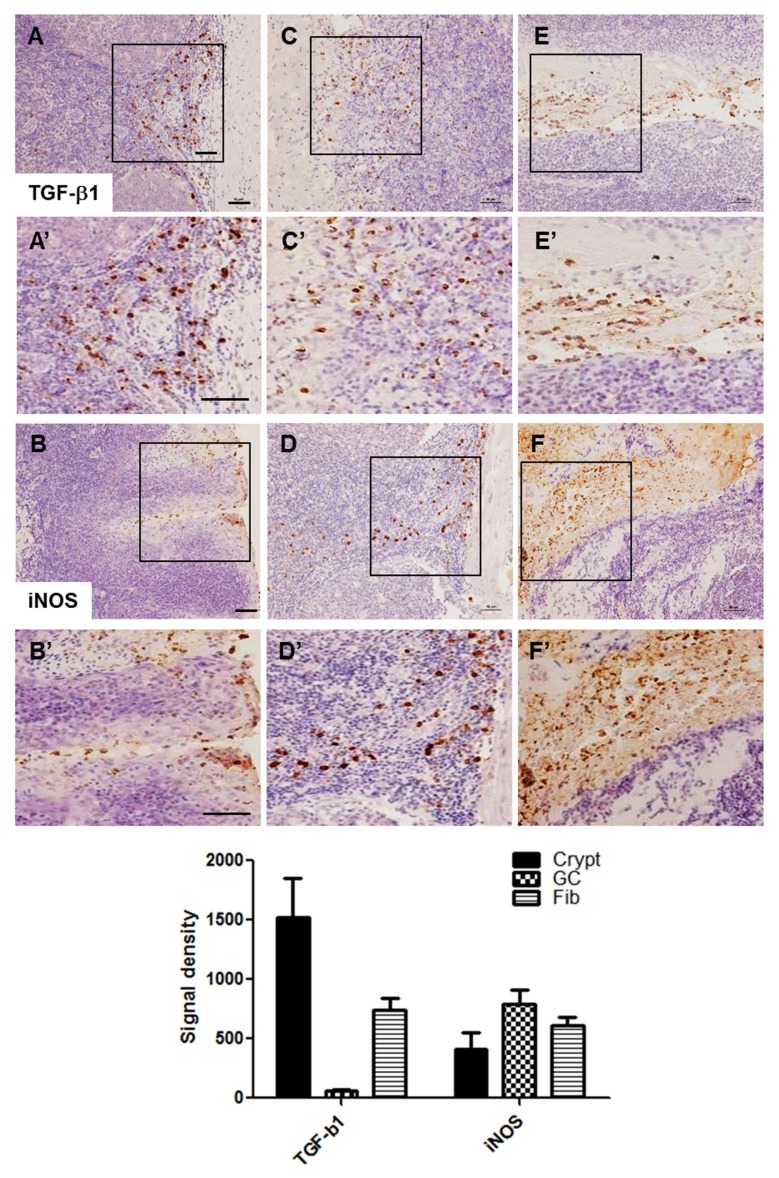
Localization of TGF- β1 and iNOS-positive cells in the tonsil sections. TGF- β1 and iNOS-positive cells were found in the crypt epithelium (A and B), surrounding germinal centers (C and D), and surrounding the lymphoid follicle with excessive developing fibrosis (E and F). Inserts in each image was magnified (A’-F’). Scale bar, 50 μm.

Immunohistochemical staining for TGF-β1 and iNOS was performed to investigate the relationship between pepsin staining and inflammation. Both TGF-β1 and iNOS-positive signals were also detected in regions similar to pepsin staining, such as in the crypt epithelium (Fig 3A and 3B), surrounding the germinal centers (Fig 3C and 3D), and surrounding the lymphoid follicle with excessive developing fibrosis (Fig 3E and 3F).

### Pepsin and TGF-β1 were detected in CD68-positive cells in the tonsil hypertrophy tissue

**Fig 4:**
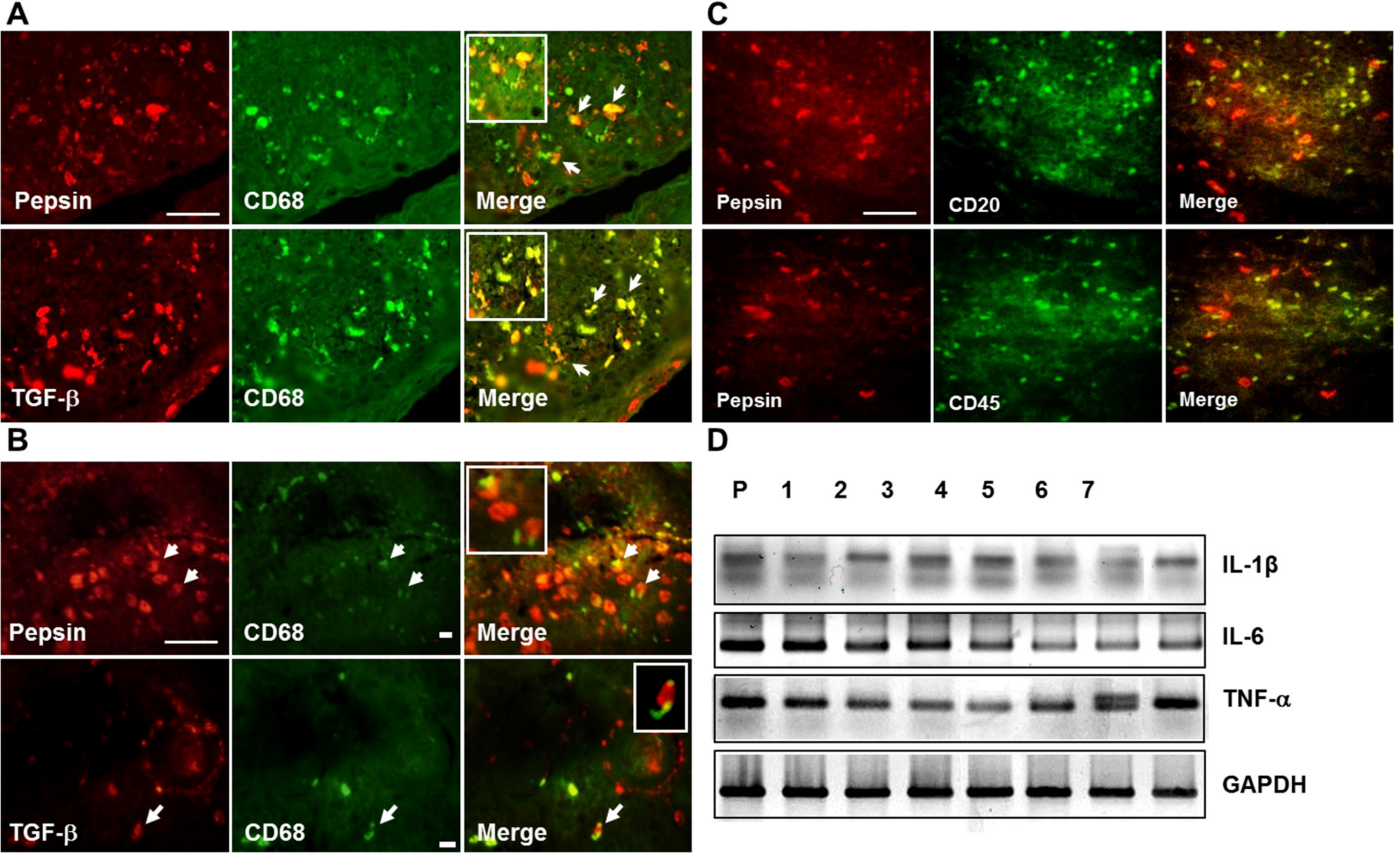
Colocalization of TGF-β1, pepsin and macrophage and lymphocyte-positive cells in the tonsil sections and mRNA expression for inflammatory mediators in tonsil tissues with tonsillar hypertrophy. **A)** Dual pepsin and CD68-positive cells and dual TGF-β1and CD68-positive cells were detected in the crypt. **B)** Dual pepsin and CD68-positive cells and dual TGF- β1and CD68-positive cells were also detected in the surrounding germinal centers. **C)** Few dual pepsin and CD20 or CD45-positive cells were detected. Scale bar, 50 μm. **D)** The mRNAs of IL-1, IL-6, and TNF- β in the tonsil tissues were determined by RT-PCR. GAPDH housekeeping gene was used as a control.

As described in Introduction, we hypothesized that pepsin staining in the tonsil originates from the stomach and could be related to tonsil inflammation. Double immunofluorescence staining for CD68 was performed to characterize the pepsin-positive cells. CD68 is a 110-Kd transmembrane glycoprotein and a representative marker of human monocytes and tissue macrophages involved in inflammation. CD68-positive cells were clearly observed in the tonsil with tonsil hypertrophy (Fig 4). Of note, CD68-positive cells strongly colocalized with pepsin and TGF- β1-positive cells both in the crypt (Fig 4A) and surrounding germinal centers (Fig 4B). In contrast to colocalization of pepsin and CD68, pepsin did not co-localize with B-lymphocytes (CD20 positive) in the follicular centers and T-lymphocytes (CD45) in the interfollicular regions (Fig 4C). These data, as well as that shown in Fig 3 suggested that pepsin staining might be related to the inflammatory responses in the tonsil of patients with tonsil hypertrophy. And also, to reveal the major mechanism of tonsil injury by inflammatory mediators mediated by white cells including PBMNCs and macrophages, levels for tonsillar IL-6, IL-1β and TNF-α mRNA were examined by RT-PCR (Fig 4D). Interestingly, all of these were expressed in the tonsil tissues with tonsillar hypertrophy. This data suggests that the major mechanism of tonsillar hypertrophy might be caused by inflammatory mediators.

### Pepsin drove the patients-derived monocytes to differentiate to macrophages

**Fig 5:**
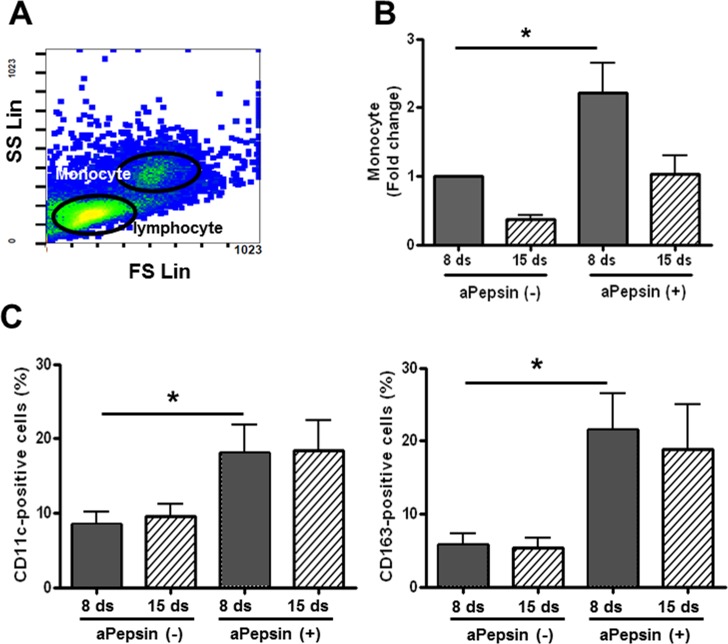
Flow cytometric analysis of monocyte population and monocyte differentiation from PBMNCs from tonsillar hypertrophy. **A)** Lymphocytes and monocytes were identified with side and forward scatter profiles. Lymphocytes and monocytes were also confirmed by staining with CD4 and CD8 and CD14 antibodies. **B)** PBMNCs were cultivated in macrophage-specific culture conditions with or without activated pepsin for 15 days. Monocyte population was identified with side and forward scatter profile on flow cytometry for each condition. Each level was based on the value from day 8 (pepsin-untreated, control cells) and given a value of “1” as baseline. **C)** Monocyte to macrophage differentiation was examined by staining with CD11c and CD163 antibodies.

To confirm the relation of pepsin staining and macrophages, we cultivated peripheral blood mononuclear cells (PBMNCs) from tonsillar hypertrophic patients in macrophage culture medium (in presence or absence of activated pepsin) for 15 days. We furthermore determined levels of monocyte population, and analyzed macrophage phenotype by flow cytometry. Human macrophages are produced by the differentiation of monocytes in tissues. They play a critical role in non-specific defense (innate immunity), and also help initiate specific defense mechanisms (adaptive immunity) by recruiting other immune cells such as lymphocytes. They can be identified using flow cytometry by their specific expression of proteins as CD markers including CD11c and CD163. The population of monocytes inferred from flow side and forward scatter (Fig 5A) was significantly increased in presence of activated pepsin (aPepsin), as compared to no increase on day 8, and no significant increase on day 15 (Fig 5B). In addition, we investigated the monocyte to macrophage differentiation using CD11c and CD163 antibodies. The CD11c and CD163-positive cells were significantly increased by aPepsin on day 8 after cultivation. No significance was found in other conditions (Fig 5C). However, the monocyte population was not significant and also levels for CD11c and CD163 in adult group both on day 8 and 15. This data suggests that pepsin is potentially involved in macrophage differentiation and children with increased stomach reflux may be more exposed to effects of a pepsin environment than adults.

### Pepsin induced macrophage viability and migration

**Fig 6:**
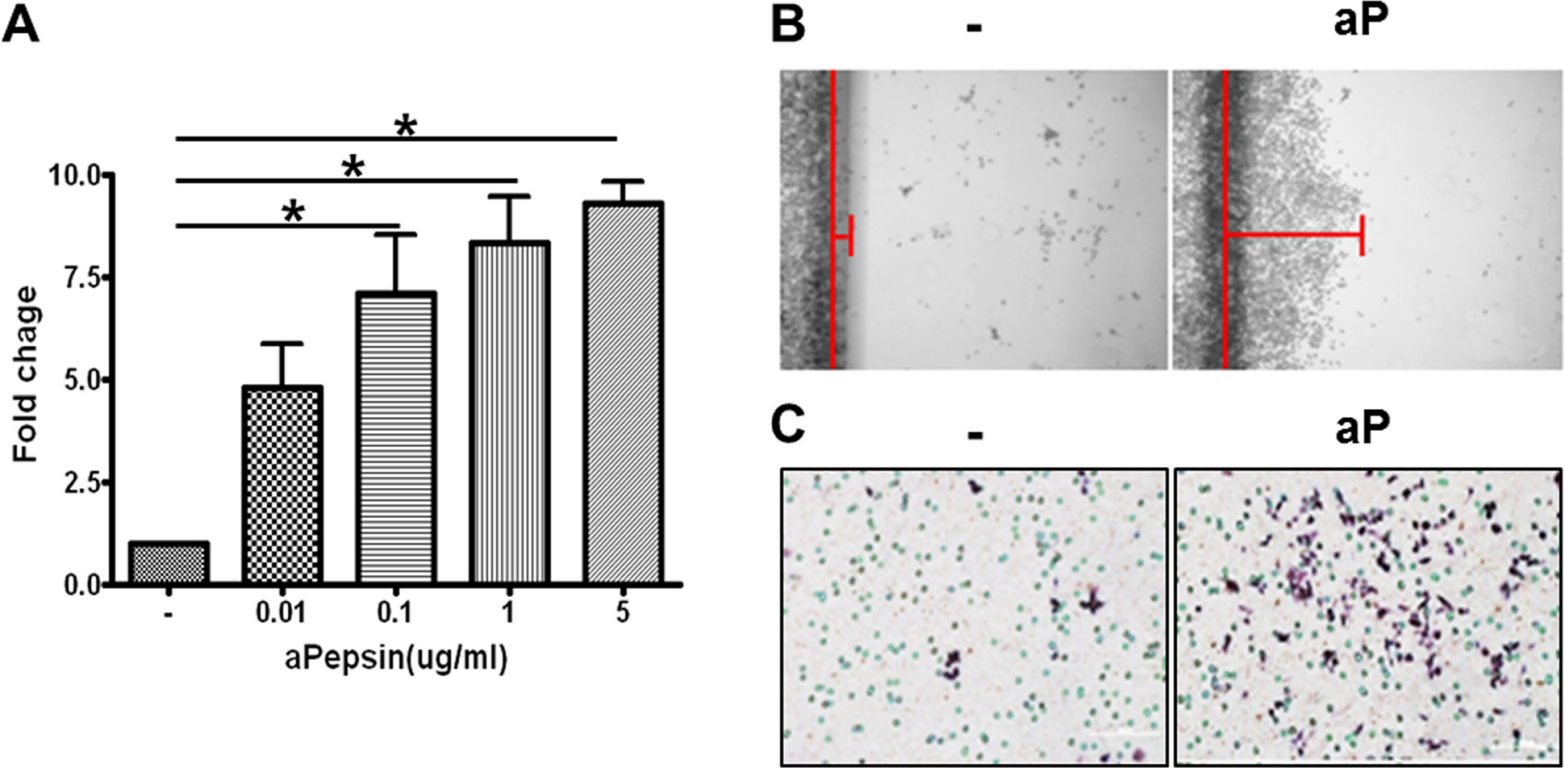
Direct effects of pepsin on macrophage viability and migration using the RAW264.7 cells. **A)** RAW264.7 cells were treated with various doses of pepsin for 24 hours. Viability was measured by WST-1 assy. The fold changes are calculated as the ratio of the final value in the each presence of pepsin to the value in absence of pepsin (set as “1”). Values are represented as the mean ± SEM. *P < 0.05 vs. the corresponding absence of pepsin (0 μg/ml). **B)** Migration of RAW264.7 cells was increased with 0.1 μg/ml of activated pepsin by scratch wound migration assay. **C)** Migration of RAW264.7 cells was also induced with 0.1 μg/ml of activated pepsin by transwell migration system.

We also investigated whether pepsin was involved in macrophage function. RAW264.7 cells were cultured in the presence or absence of activated pepsin for 24 hours. There was a significant dose-dependent increase in RAW264.7 cell viability by pepsin (Fig 6A). Migration of RAW264.7 cells was also induced by pepsin in both scratch wound and transwell migration system assays (Fig 6B and 6C).

## Discussion

This study first showed that pepsin was detected in the hypertrophic tonsil and pepsin-positive cells were localized in the crypt epithelium surrounding the germinal center, and in the lymphoid follicle with excessive developing fibrotic appearance. Notably, pepsin staining was correlated with expression of inflammation-related factors, and pepsin and CD68 colocalized, and activated pepsin led to differentiation of monocytes to macrophages.[16] These findings point to potentially novel pathophysiological mechanisms underlying tonsillar hypertrophy.

Intense inflammation is a known risk factor for tonsil hypertrophy.[17] TGF-β1 and iNOS are known mediators of inflammation.[18–20] In hypertrophic tonsils, the increase in T and B cell counts showed a positive correlation with bacterial counts and tonsil size.[21, 22] In epidemiologic studies, smoking, allergies and recurrent respiratory infections might associate with transient or permanent hypertrophy of lymphoid tissue.[22, 23] Immunologic parameters, genetic predisposition and local lymphocyte dysfunction appear to play a role in the etiology of recurrent tonsillitis and tonsillar hypertrophy.[22, 24] Some studies demonstrated that tonsillar hypertrophy was associated with increased lymphoid follicle size, but not the number of follicles[25] and was also related to increased tonsil weight, increased follicle diameter, area and number.[26]

**Fig 7:**
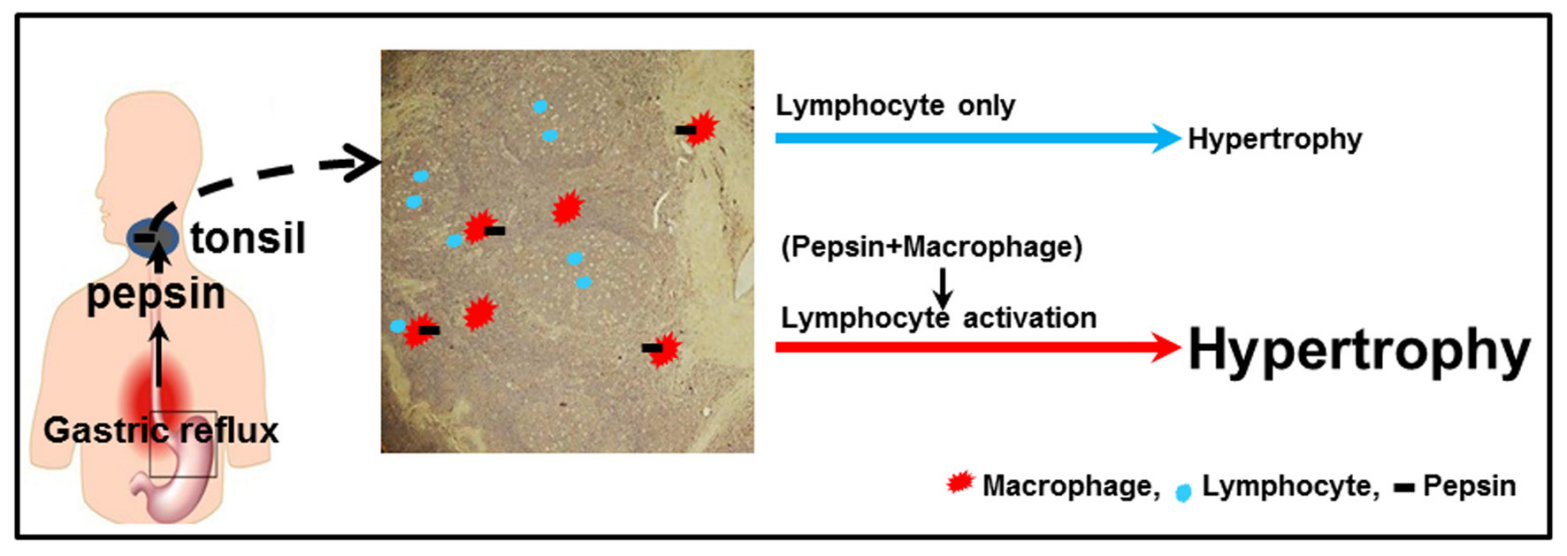
Two hypotheses for pepsin-mediated tonsillar hypertrophy.

Recurrent stimuli by pathogenic agents, during the inflammatory process, lead to activation of monocytes and macrophages.[27] The cytokines secreted by macrophages stimulate immune cells, and also cause proliferation of endothelial cells and fibroblasts.[28] With time, an immunologically active tissue is replaced by fibrotic tissue.[28] In this study, we assumed that the antigen was pepsin and we put forward two hypotheses to explain the observation of tonsillar hypertrophy with gastric reflux (Fig 7). One mechanism could be direct stimulation of the lymphocytes by pepsin of refluxate that are recognized as antigenic. Another possible mechanism involves pepsin-induced injury to the epithelium in the tonsillar crypts, which results in cryptitis from resident bacteria with ongoing antigenic stimulation of the specialized crypt epithelium. These would lead to an increase in the number of lymphocytes and may play a role in tonsillar hypertrophy.

Initially pepsin comes into contact with the epithelium and is presented to the intraepithelial lymphocytes, the subepithelial lymphocytes, interfollicular and intrafollicular lymphocytes, in that order. The lymphocytes then proliferate in response to pepsin acting as an antigen, causing the tonsillar follicles to enlarge and the tonsil tissues to undergo hypertrophy. Alternatively, tonsil-tissue macrophages recognize the reflux-mediated pepsin on their cell surface as a foreign body and are activated. Macrophage activation causes secretion of pro-inflammatory cytokines, and these cytokines induce inflammation as well as additional lymphocyte activation, that result in tonsillar hypertrophy. This latter hypothesis appears to have more support than the former since as shown in Fig 4, pepsin and CD68-positive cells were colocalized below the surface epithelium located in the crypt (Fig 4A) and also surrounding the lymphoid follicle with excessive ensuing fibrosis (Fig 4B). However, little correlation with pepsin and CD20 and CD45, as markers of B and T cells respectively, was observed in the tonsil tissue (Fig 4C).

We also assessed the expression of CD163 from PBMNCs culture with tonsillar hypertrophy. CD163 is expressed only by mature macrophages, but absent on monocytes. We cultured PBMNCs for 8 and 15 days in the presence of 10% FCS and 10 ng/ml M-CSF, following standard conditions for the culture of human macrophages. Results of *in vitro* PBMNCs culture studies showed that in the child group, CD163-positive cell numbers were significantly higher in presence of activated pepsin as well CD11c-positive cells. In comparison, there was no difference in the expression of CD11c and CD163 from the culture of PBMNCs from adults (S1 Fig). These data suggested that a PBMNC reaction to activated pepsin in children might be more sensitive than in adults. Although we cannot explain which pepsin-induced mechanism is involved in macrophage differentiation, we cannot rule out that macrophage differentiation itself could accelerate reflux-mediated tonsil damage.

According to the current study, pepsin-mediated reflux cause not only direct damage to the tonsillar epithelium, but also stimulated the tonsillar macrophages or tonsillar epithelial cells to secrete chemokines/cytokines that attracted and activated the immune cells that mediated some of the damage to the tonsil mucosa. Microscopic inflammation, characterized by TGF- β1 and iNOS expression in the tonsil tissue (crypt epithelium, surrounding the germinal center, and the lymphoid follicle with excessive developing fibrotic appearance), is observed in patients with severe symptoms (data not shown). This implies that the pepsin (and acid)-induced production of IL-8 and other inflammatory mediators by the refluxate promote migration and activation of peripheral blood leukocytes.[14] These findings corroborate the hypothesis that a cytokine-mediated mechanism is responsible for the tonsil injury in children with tonsillar hypertrophy. The mucosa of patients with tonsillar hypertrophy produces significantly large amounts of various cytokines.[29, 30] These inflammatory mediators activate immune cell recruitment and migration to the sites of refluxate interaction and may be involved in the pathophysiology of tonsil hypertrophy.

Based on the findings from the literature and our results in this study, we propose that local and systemic activation of inflammatory pathways will promote lymphocyte infiltration and proliferation (including T cells) along with macrophage differentiation and proliferation resulting in tonsillar hypertrophy from the increased monocytic and lymphocytic cell numbers. If the current findings prove to be accurate, they may provide a viable target for development of interventional approaches for treatment or prevention of tonsillar hypertrophy in children. Despite the considerable evidence for inflammatory mediators and lymphocyte proliferation in the pathogenesis of tonsil hypertrophy, the interplay between hypersensitivity to pepsin reflux and tonsillar inflammation remains unclear in this study. Further studies are warranted to better understand the signaling pathways involved in the genesis of reflux symptoms and inflammation and to identify along with developing new therapeutic approaches.

## Conclusions

We established that lymphocytes and monocytes are in a highly proliferative state in the tonsils with tonsillar hypertrophy and associated with increased expression of pro-inflammatory factors as a result of exposure to gastric reflux pepsin. These findings point to potentially novel pathophysiological mechanisms underlying tonsillar hypertrophy. From our *in vitro* data, we establish that there may be the possibility of objective characterization of the mechanisms involved in order to develop specific treatments for this disease indication. Our data suggested that the mechanisms underlying lymphoid tissue proliferation in tonsillar hypertrophy are distinct and may allow for future non-surgical therapeutic interventions targeting pepsin that may obviate the need for a tonsillectomy, and leading to involution of the hypertrophic tonsils.

## Supporting Information

S1 Fig Flow cytometric analysis of monocyte population and monocyte differentiation from PBMNCs of adults with chronic tonsillitis. Lymphocytes and monocytes were identified with side and forward scatter. Lymphocytes and monocytes were also confirmed by staining with CD4 and CD8 and CD14 antibodies. PBMNCs were cultivated in macrophage-specific culture conditions with or without activated pepsin for 15 days. Monocytes population was identified from side and forward scatter profiles in flow cytometry in each condition. Each level was compared with the value of day 8 cells in absence of pepsin that were given an arbitrary value of “1”. Monocyte to macrophage differentiation was examined by staining with CD11c and CD163 antibodies.
(TIF)
