# Supplementary figures and images for: Extra-Esophageal Pepsin from Stomach Refluxate Promoted Tonsil Hypertrophy
Source: PLoS One. 2016 Apr 8;11(4):e0152336. doi: 10.1371/journal.pone.0152336 (PMC4825923; doi:10.1371/journal.pone.0152336)

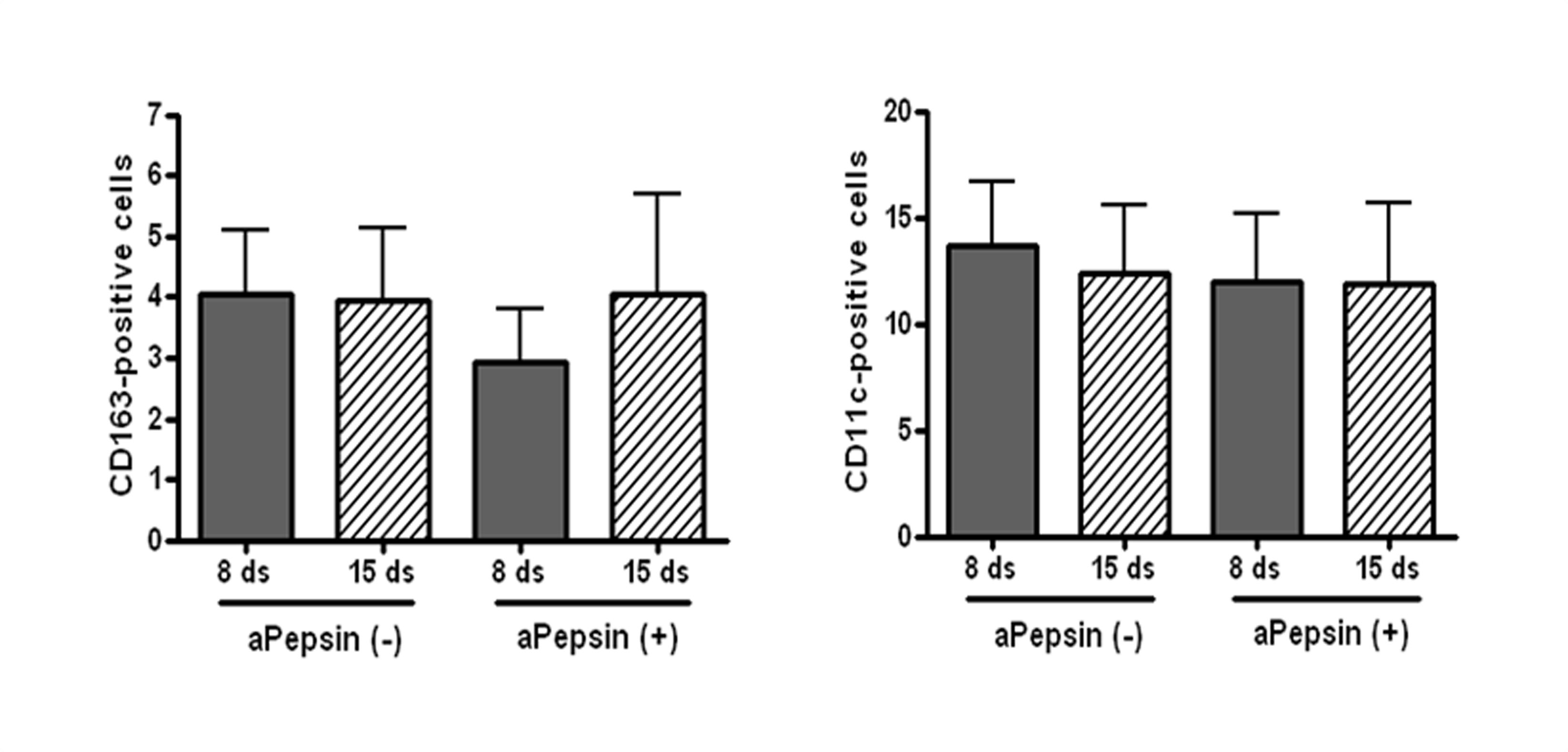

Supplement: S1 Fig — Lymphocytes and monocytes were identified with side and forward scatter. Lymphocytes and monocytes were also confirmed by staining with CD4 and CD8 and CD14 antibodies. PBMNCs were cultivated in macrophage-specific culture conditions with or without activated pepsin for 15 days. Monocytes population was identified from side and forward scatter profiles in flow cytometry in each condition. Each level was compared with the value of day 8 cells in absence of pepsin that were given an arbitrary value of “1”. Monocyte to macrophage differentiation was examined by staining with CD11c and CD163 antibodies. (TIF) [file pone.0152336.s001.tif]
